# Pre-treatment Serum HE4 Level as a Novel Independent Prognostic Biomarker for Uterine Cervical Carcinoma Patients

**DOI:** 10.3389/fonc.2020.584022

**Published:** 2020-09-29

**Authors:** Eliana Bignotti, Laura Zanotti, Paola Todeschini, Valentina Zizioli, Chiara Romani, Davide Capoferri, Germana Tognon, Enrico Sartori, Stefano Calza, Franco Odicino, Antonella Ravaggi

**Affiliations:** ^1^Division of Obstetrics and Gynecology, Azienda Socio Sanitaria Territoriale (ASST) Spedali Civili di Brescia, Brescia, Italy; ^2^“Angelo Nocivelli” Institute of Molecular Medicine, University of Brescia and ASST-Spedali Civili of Brescia, Brescia, Italy; ^3^Department of Clinical and Experimental Sciences, Division of Obstetrics and Gynecology University of Brescia, Brescia, Italy; ^4^Unit of Medical Statistics, Department of Molecular and Translational Medicine, University of Brescia, Brescia, Italy; ^5^Department of Medical Epidemiology and Biostatistics, Karolinska Institute, Stockholm, Sweden; ^6^Big & Open Data Innovation Laboratory (BODAI) Lab, University of Brescia, Brescia, Italy

**Keywords:** uterine cervical carcinoma, HE4, biomarker, prognosis, serum

## Abstract

In spite of the effective implementation of screening programs, uterine cervical carcinoma (UCC) remains one of the major causes of cancer death among women around the world. The aim of this study was to investigate the prognostic value of serum human epididymis protein 4 (HE4) in UCC. Pre-treatment serum samples from 109 UCC patients and 99 healthy women were analyzed for HE4 levels by a quantitative chemiluminescent microparticle immunoassay on the automated ARCHITECT instrument. HE4 serum (sHE4) levels were significantly higher in UCC patients, regardless of tumor stage, compared with healthy controls. Elevated sHE4 levels were significantly associated with advanced FIGO stage and absence of disease-free interval after treatment. In univariable analysis, higher sHE4 levels were significantly correlated with shorter overall survival and progression-free survival. In multivariable analysis, sHE4 retained its significance as independent adverse prognostic factor for both survival endpoints. This study indicates that sHE4 is associated with a more aggressive tumor phenotype and a worse patient's prognosis. These results suggest the potential role of sHE4 as a novel prognostic marker and as an indicator of high-risk UCC patients for a tailored surgical and adjuvant therapy.

## Introduction

Uterine cervical carcinoma (UCC) is one of the most common gynecological malignant neoplasms worldwide, with about 80% of cases occurring in developing countries. The estimated global number of new UCC patients reaches up to 466,000 and ~270,000 women are expected to die from the disease annually (1, 2). Although treatment efficacy of conventional treatment strategies including radical surgery, radiotherapy, and chemotherapy, and, more recently, targeted therapy has significantly improved, the clinical outcome of UCC patients still remains dismal, with a median overall survival for advanced cases of 16.8 months (3). Almost 20% of early-stage UCC patients will experience relapse, while the recurrence rate of patients with advanced-stage raises up to 70% (4). Moreover, UCC patients sharing similar clinical and pathological characteristics can show a variable relapsing interval as well as variable survival rates, suggesting that current clinical tools are not sufficiently adequate to reliably predict patient outcome. The correct classification of UCC patients in different prognostic classes is essential in order to decide the timely, appropriate, and effective therapy and to control either disease recurrence or to improve quality of life.

Serum biomarkers, holding the potential of cost-effectiveness, non-invasivity, and reproducibility, are often used in clinical practice for early tumor detection, response to treatment monitoring and prognosis prediction (5). Several biomarkers with potential prognostic role have been identified in UCC (6, 7), but none of them has been introduced in clinical practice so far. Among recently reported serum biomarkers in gynecological cancer, one of the most promising is the Human Epididymis Protein 4 (HE4 or WFDC2), a member of the whey acidic protein (WAP) four-disulfide core gene cluster bearing a conserved motif found in several protease inhibitors (8). HE4 was first described by Kirchhoff et al. (9) in human epididymal tissue and was subsequently found expressed in many other normal tissues, particularly of the reproductive tracts and of the central respiratory airways, as well as in gynecological malignancies, where HE4 has probably shown the most promising clinical results for early diagnosis and prognosis (10–15). Conversely, data regarding its role in UCC are lacking.

Herein, we have investigated pre-treatment sHE4 levels in a cohort of 109 UCC patients and 99 healthy women and we have correlated them with patients' clinicopathological features and survival endpoints to determine its prognostic value.

## Materials and Methods

### Patients' Characteristics and Sample Collection

The 109 serum samples included in this investigation were collected from sequential UCC patients treated at the Division of Obstetrics and Gynecology, ASST Spedali Civili of Brescia, Italy from 2003 to 2013. Control sera were collected from 99 women consecutively enrolled from 2003 to 2010, referred to the same Institution for routine gynecological exams or for uterine prolapse surgery. These healthy controls were characterized by the absence of benign or malignant gynecologic disorders, assessed by medical history, gynecological examination, pelvic transvaginal ultrasound, and PAP-smear test. All subjects with a past or concomitant history of malignancy, and patients with renal failure or with creatinine >1.5 mg/dl were excluded from the study, due to the high levels of non-UCC-related sHE4 in these patients (16). The present investigation was performed in accordance with the ethical standards of the European Union and with the 1964 Helsinki declaration and its later amendments, and approved by the Research Review Board—the Ethic Committee—of the ASST Spedali Civili, Brescia, Italy (study reference number: NP1545). Written informed consent was obtained from each patient prior to blood withdrawal. Fasting blood samples were collected from patients before primary treatment (surgery or chemo-radiation therapy), hereinafter called “pre-treatment.” Sera were separated within 1 h by centrifugation at 1,500 g for 10 min, and then stored at −80°C until analysis. UCC patients' charts were reviewed to obtain all clinical and pathological features at diagnosis and during follow-up. UCC patients were staged in accordance with International Federation of Gynecologists and Obstetricians (FIGO) guidelines issued in 2009. Clinicopathologic characteristics of UCC patients are summarized in Table 1. UCC patients and controls were well-balanced with respect to age (median 48, range 23–87 for UCC patients; median 54, range 21–66 for controls; *t*-test *p* = 0.996). The study included patients with histologically confirmed UCC of different histological type and FIGO stage I–IV. Fifty-eight out of 109 patients (53%) underwent upfront surgery and 32 of them received an adjuvant treatment: radiotherapy (18/32), platinum-based chemotherapy (5/32), or both (9/32). Forty-one out of 109 patients (38%) received platinum-based neoadjuvant chemotherapy, followed by surgery for 23 of them or radiotherapy for 18 of them. Seven out of 109 (6%) were treated exclusively with chemotherapy and three out of 109 patients (3%) received only radiotherapy.

**Table 1:** Clinicopathological characteristics of UCC patients and association with pre-treatment sHE4 levels.

	**sHE4 pmol/L**				
**Variables**	**No. of patients (%)**	**Geometric mean (SD)**	**Median (IQR)**	**FC (CI_95%_)**	***p*-value**
**All patients**	109 (100%)	56.3 (2.02)	46.7 (28.8)	–	
**Age (years)**					0.089
<48	47 (43%)	45.5 (1.62)	44.8 (20.8)	1	
≥48	62 (57%)	66.2 (2.22)	51.3 (46.6)	1.16 (0.98; 1.37)	
**Histological type**					
Squamous carcinoma	73 (67%)	55.7 (1.95)	47.7 (31.4)	1	0.609
Adenocarcinoma	24 (22%)	64.4 (2.51)	45.4 (33.4)	0.92 (0.73; 1.16)	
Adenosquamous carcinoma	12 (11%)	45.8 (1.25)	45.4 (15.6)	0.91 (0.67; 1.23)	
**Grading WHO**					
G1	10 (9%)	40.6 (1.25)	39.9 (10.5)	1	0.271
G2	34 (31%)	51.2 (1.60)	44.6 (24.5)	1.17 (0.85; 1.61)	
G3	55 (51%)	56.9 (2.13)	48.0 (30.3)	1.24 (0.91; 1.68)	
Unknown	10 (9%)	101.0 (2.85)	96.6 (117.0)	–	
**FIGO stage**					
I	67 (61%)	45.2 (1.54)	44.0 (21.3)	1	** <0.001**
II	26 (24%)	50.2 (1.51)	47.4 (18.2)	1.06 (0.87; 1.28)	
III	6 (6%)	128.0 (1.93)	135.0 (49.3)	3.04 (2.13; 4.35)	
IV	10 (9%)	199.0 (3.00)	131.0 (362.0)	2.37 (1.78; 3.14)	
**Clinical tumor size**					
≤ 4 cm	49 (45%)	43.4 (1.51)	41.1 (22.3)	1	0.090
>4 cm	35 (32%)	63.6 (1.88)	48.0 (59.8)	1.18 (0.98; 1.41)	
Unknown	25 (23%)	79.1 (2.76)	52.7 (55.3)	–	
**Lymph-vascular space invasion**					
Absent	17 (16%)	42.2 (1.70)	43.5 (22.0)	1	0.338
Present	37 (34%)	43.9 (1.38)	41.1 (16.6)	0.91 (0.76; 1.10)	
Unknown	55 (50%)	72.7 (2.30)	55.7 (70.8)	–	
**Stromal Infiltration**					
<3 mm	5 (5%)	48.7 (1.28)	51.2 (15.3)	1	0.425
≥3 mm	48 (44%)	41.9 (1.51)	39.0 (17.6)	0.86 (0.59; 1.25)	
Unknown	56 (51%)	73.4 (2.27)	56.2 (66.2)	–	
**Parametrial invasion**					
Absent	47 (43%)	42.0 (1.51)	39.2 (21.5)	1	0.261
Present	9 (8%)	48.3 (1.24)	47.7 (8.0)	1.13 (0.91; 1.42)	
Unknown	53 (49%)	74.8 (2.31)	56.7 (76)	–	
**Vaginal invasion**					
Absent	43 (39%)	41.3 (1.52)	39.2 (13.3)	1	0.071
Present	14 (13%)	48.7 (1.28)	49.8 (17.9)	1.18 (0.99; 1.41)	
Unknown	52 (48%)	75.6 (2.32)	56.7 (76.5)	–	
**Lymph nodes status**					
Negative	39 (36%)	43.6 (1.53)	40.3 (23.0)	1	0.957
Positive	18 (16%)	43.9 (1.35)	45.2 (14.1)	1.00 (0.84; 1.20)	
Unknown	52 (48%)	74.4 (2.34)	56.2 (77.4)	–	
**Treatment**					
Surgery	26 (24%)	40.4 (1.58)	38.4 (24.6)	1	** <0.001**
Surgery + RT	18 (16%)	45.7 (1.39)	44.0 (18.9)	1.05 (0.75; 1.47)	
Surgery + CT	5 (5%)	40.9 (1.18)	37.8 (12.7)	0.97 (0.57; 1.64)	
Surgery + CT + RT	9 (8%)	49.6 (1.44)	52.0 (26.0)	1.22 (0.80; 1.86)	
NACT + Surgery	23 (21%)	52.4 (1.49)	47.7 (14.2)	1.17 (0.85; 1.59)	
CT	7 (6%)	194.3 (2.89)	135.4 (443.5)	3.40 (2.14; 5.39)	
NACT + RT	18 (16%)	86.2 (2.59)	83.4 (110.5)	1.82 (1.31; 2.54)	
RT	3 (3%)	63.9 (1.88)	84.0 (68.8)	1.72 (0.89; 3.34)	
**Persistence of disease**					
No	83 (76%)	46.9 (1.55)	44.4 (22.0)	1	** <0.001**
Yes	25 (23%)	101.0 (2.81)	89.7 (96.4)	1.48 (1.22; 1.80)	
Unknown	1 (1%)	112.0 (–)	112.0 (–)		

*Results are reported as estimated Fold Change (FC) from robust linear models with asymptotic CI_95%_*.

*Robust F-test p-value; Bold numbers indicate a statistically significant difference with a p < 0.05*.

*CT, chemotherapy; RT, radiotherapy; NACT, neoadjuvant-CT*.

### HE4 Serum Levels Measurement

HE4 levels of pre-treatment sera were measured using chemiluminescent magnetic microparticle immunoassay (CMIA) on the fully automated Architect instrument (Abbott Diagnostics Division, Wiesbaden, Germany). The dynamic range of HE4 detection spans from 20 to 1,500 pM, with an automated 1:10 dilution protocol that extends the linear range up to 15,000 pM. The intra-assay and total imprecision (CV %) of the CMIA HE4 assay ranged from 2.11 to 2.93% and from 3.13 to 3.70%, depending on the concentrations of the assays' positive controls. HE4 level measurement was carried out at the Clinical Chemistry Laboratory, ASST Spedali Civili of Brescia, following the manufacturer's recommendations.

### Statistical Analysis

The association between sHE4 levels and clinicopathological parameters, as well as differences between UCC patients and controls, were evaluated using robust linear models (17). Area under the receiver operating characteristic (ROC) curve was used to quantify sHE4 ability to discriminate between the UCC and healthy controls. Due to the highly positively skewed distribution, sHE4 was transformed on log_2_ scale. For survival analyses, Progression-Free Survival (PFS), and Overall Survival (OS) were considered as endpoints. PFS was defined as the time interval between the date of diagnosis and the date of identification of disease recurrence, in patients who completely responded to treatment, or of a progressive disease (disease not treatable with curative intent) in those patients who never achieved complete remission. Notably, we could identify an earlier progression event for all subjects who died of disease. OS was defined as the time interval between the date of diagnosis and the date of death or the last follow-up. For both endpoints, the last date of follow-up was used for censored subjects. Median follow-up time was computed using Kaplan–Maier method applied to the censored times reversing the roles of event status and censored. Survival analyses were performed using the Cox proportional hazard models. The assumption of proportionality of hazards was checked (18). Variable selection in the multivariable model was performed both using backward selection and LASSO (19). Briefly, we fitted models accounting for HE4 (log_2_ scale), stage, age, treatment, and kept only those variables with a non-null parameter estimates. Penalization parameter (lambda) tuning was performed using cross-validation. The existence of a non-linear relationship between OS or PFS and sHE4 was evaluated fitting sHE4 with restricted cubic splines (20) and different models compared using both the Akaike information criterion (AIC) and likelihood ratio tests (LRT). Due to the substantial number of missing values, clinically relevant variables such as lymph nodes status, stromal infiltration, parametrial invasion, and tumor volume were not considered in the main survival analysis. Nevertheless, to evaluate their effects on sHE4 effect estimates, we first imputed missing data using “MICE” algorithm (21) (*m* = 9, number of multiple imputations). Variable selection and model fitting were performed on every imputed dataset as described above, and covariate with a non-zero coefficient in more than 50% of the imputed dataset were kept in the final model. Global estimates are then computed pooling estimates from every imputed dataset. The relationship between continuous sHE4 values and survival was described using the Kaplan–Meier method. In this regard, we estimated a cut-off using maximally selected statistic (22) and the estimated survival curves were labeled as low and high levels of the marker. All statistical tests were two sided and assumed a 5% significant level. Statistical analyses were performed using R (version 4.0.0, R Development Core Team, 2010) and SPSS Statistics (Version 23.0).

## Results

### sHE4 Levels in Tumors vs. Negative Controls

**Figure 1:**
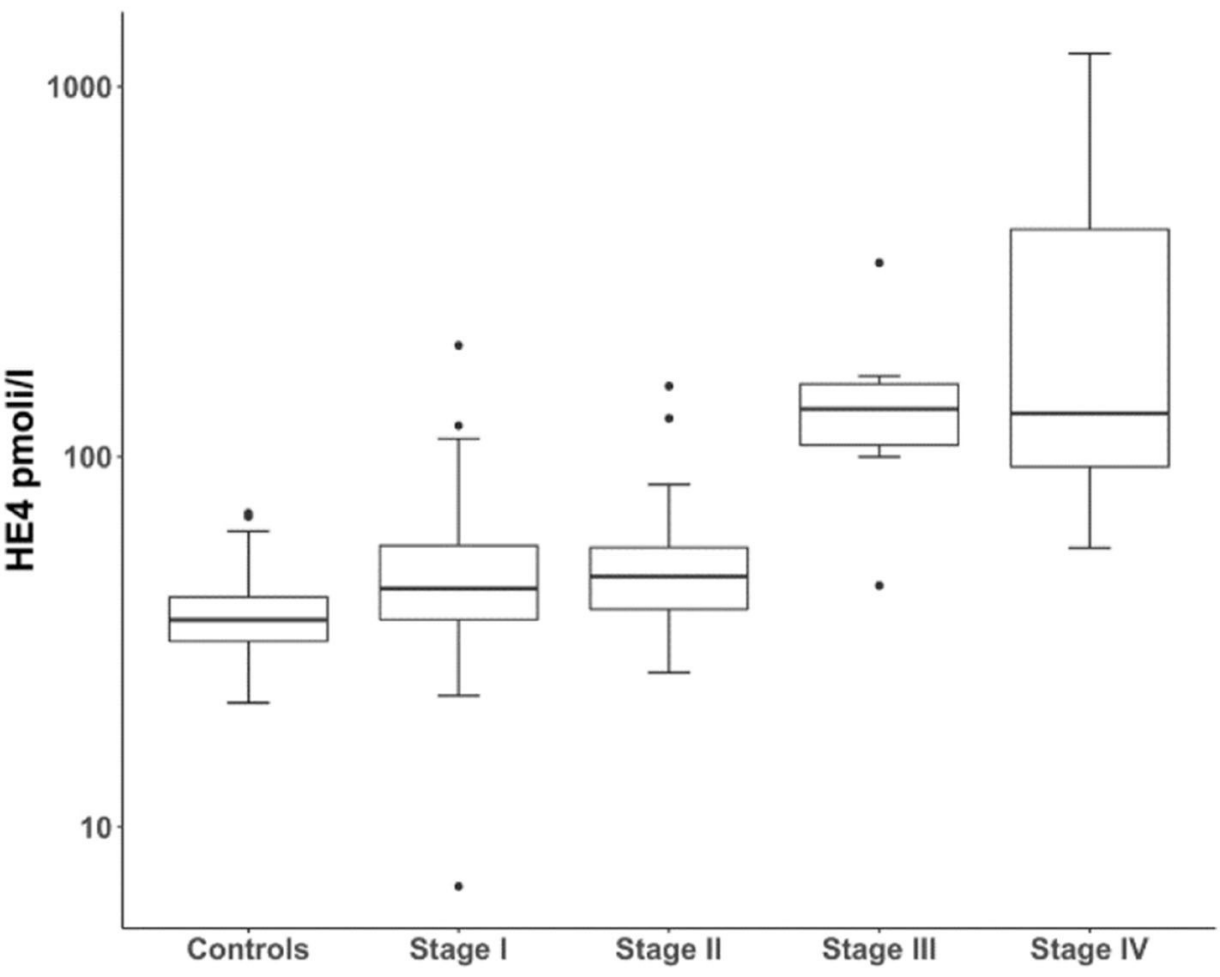
Boxplots of sHE4 levels in normal controls and UCC patients, according to FIGO stage.

sHE4 concentration was significantly higher in UCC patients, regardless of tumor stage, compared with negative controls (Stage = 1 vs. Controls, FC = 1.23, CI_95%_ = 1.09–1.38, *p* < 0.001; Stage > 1 vs. Controls, FC = 1.49, CI_95%_ = 1.31–1.70, and *p* < 0.001; Figure 1). Supplementary Table 1 shows all the sHE4 values obtained in the 208 samples analyzed.

The overall ability of sHE4 to discriminate between healthy subjects and UCC patients was evaluated by a ROC analysis, that reached an area under the curve (AUC) value of 0.754 (CI_95%_ = 0.689–0.819, *p* < 0.001) (Supplementary Figure 1). Using a cut-off of 42.9 pmol/L (corresponding to the Youden Index), the sensitivity, specificity, and positive and negative predictive values of sHE4 in discriminating UCC patients from normal controls were 63.3, 79.8, 77.5, and 66.4%, respectively.

### sHE4 Levels and Correlation With Clinicopathological Variables

Table 1 shows pre-treatment sHE4 levels in UCC patients according to clinicopathological characteristics. Lymph-vascular space invasion, stromal infiltration, parametrial and vaginal invasion, and lymph node status were evaluated only in the 58 UCC patients undergoing upfront surgery. High sHE4 concentration was significantly associated with advanced FIGO stage (*p* < 0.001) and persistence of disease after primary treatment (patients with persistence of disease vs. patients with a disease-free interval, *p* < 0.001). sHE4 levels were marginally associated with age (*p* = 0.089), tumor size (*p* = 0.090), and vaginal invasion (*p* = 0.071), while no significant association was found with histological type, stromal infiltration, lymph-vascular space invasion, parametrial invasion, tumor grade, and lymph node involvement.

### sHE4 Levels and Clinical Outcome

**Figure 2:**
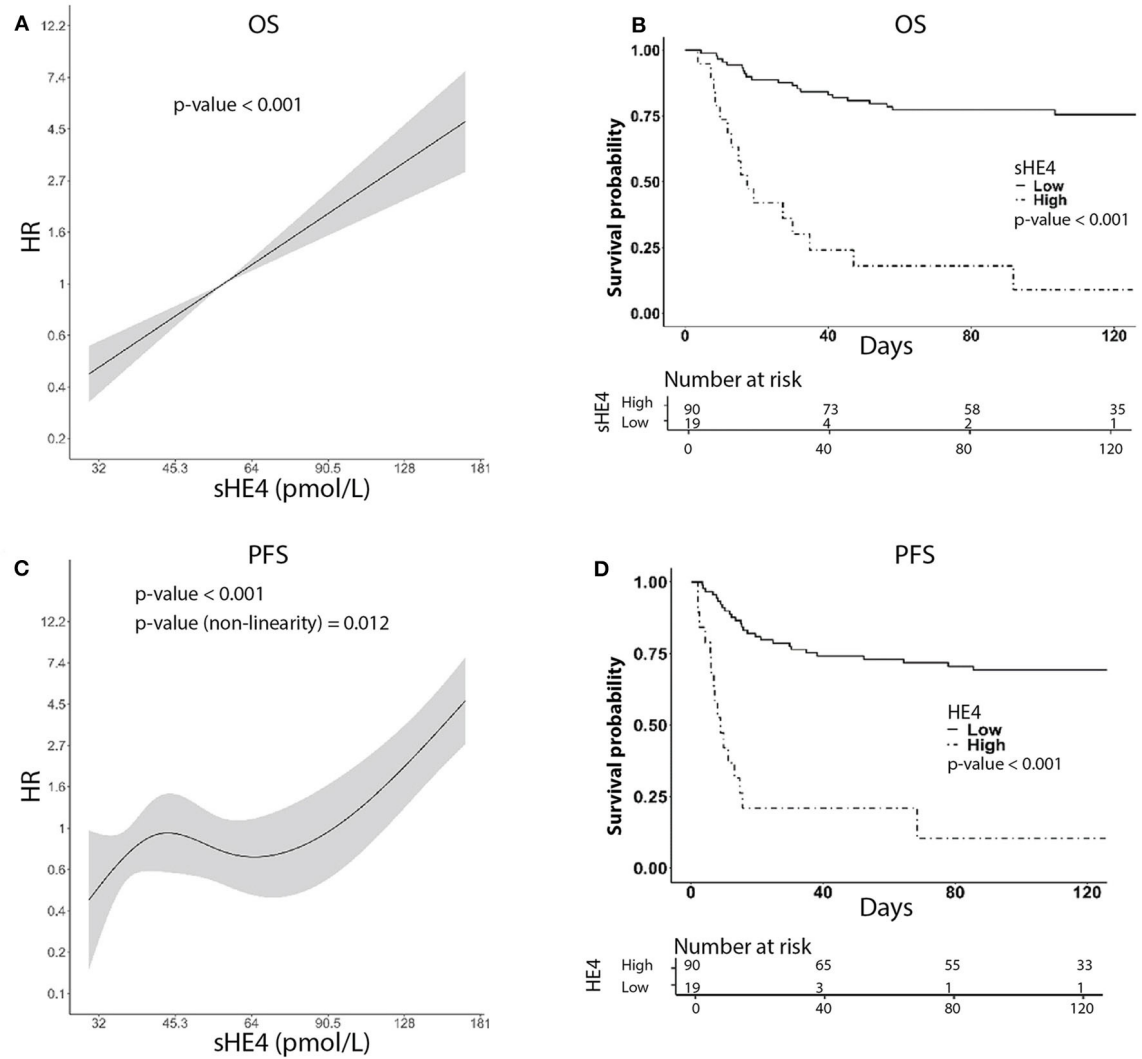
Univariable survival curves using HE4 as predictor. **(A,C)** Show HR as a function of HE4 levels for OS and PFS, respectively. **(B,D)** Show Kaplan–Meier curves, respectively, for OS and PFS, derived from sHE4 levels categorization based on cut-point estimation via generally maximally selected statistic. For illustration purposes, we label the estimates as low and high levels of HE4.

For survival analysis, patients were followed from the date of diagnosis until death or until the day of the last observation (median follow-up time: 122 months, CI_95%_ = 103–134, and range 3–162 months). At the last check, 67 patients were alive without disease, four patients were alive with disease, 37 patients died of the disease, and one patient died of another cause. In univariable analysis, higher sHE4 levels were significantly associated with poorer OS (*p* < 0.001) and shorter PFS (*p* < 0.001; Table 2), as well as other clinicopathological parameters such as higher age, advanced FIGO stage, larger tumor size, parametrial invasion, lymph node metastasis, and type of primary treatment (chemo-radiation compared to surgery) (Table 2). Notably, PFS and sHE4 (on log_2_ scale) showed a significant non-linear relationship (LRT, *p* = 0.011) that was modeled using a restricted cubic spline. For graphical representation, we described the relationship between continuous sHE4 values and survival using Kaplan–Meier estimator. Both for OS and PFS, the optimal cut off was 84 pmol/L corresponding to the 82.5% percentile of the sHE4 distribution (Figure 2).

**Table 2:** Univariable and multivariable Cox models for OS and PFS according to clinical parameters and sHE4 levels of UCC patients.

	**No. of patients**	**No. of events**		**OS**			**PFS**		
		**OS**	**PFS**	**HR**	**CI95%**	***p*-value**	**HR**	**CI95%**	***p*-value**
**Age (years)**	109	37	43	1.05	1.02–1.08	<0.001	1.05	1.02–1.08	<0.001
**Histological type**									
SCC	73	28	32	1					
Non-SCC	36	9	11	0.61	0.29–1.30	0.200	0.66	0.33–1.31	0.236
**Grading WHO**									
G1/G2	44	11	13	1					
G3	55	20	23	1.73	0.83–3.61	0.145	1.72	0.87–3.40	0.117
**FIGO stage**									
I	67	9	14	1					
>I	42	28	29	7.90	3.70–16.88	** <0.001**	5.49	2.88–10.47	** <0.001**
**Clinical tumor size**									
≤ 4 cm	49	4	8	1					
>4 cm	35	19	21	8.46	2.87–24.97	** <0.001**	4.98	2.20–11.29	** <0.001**
**Lymph-vascular space invasion**									
Absent	17	3	5	1					
Present	37	4	6	0.60	0.14–2.70	0.508	0.51	0.16–1.68	0.270
**Stromal Infiltration**									
<3 mm	5	0	1	–					
≥3 mm	48	4	8	–	–	0.500^$^	0.76	0.10–6.12	0.800
**Parametrial invasion**									
Absent	47	3	7	1					
Present	9	3	4	6.34	1.28–31.55	**0.024**	3.59	1.05–12.3	**0.042**
**Vaginal invasion**									
Absent	43	4	7	1					
Present	14	3	5	2.62	0.59–11.73	0.207	2.71	0.86–8.54	0.090
**Lymph nodes status**									
Negative	39	1	5	1					
Positive	18	6	7	15.28	1.84–127.0	**0.012**	3.65	1.16–11.52	**0.027**
**First line treatment**									
Surgery	58	7	12	1					
Chemo/radio	51	30	32	7.26	3.17–16.64	** <0.001**	4.61	2.36–9.03	** <0.001**
**log**_**2**_ **HE4**	109	37	43	2.70	1.98–3.68	** <0.001**	2.98	1.99–4.45^*^	** <0.001**
increase									
**Multivariable models**									
**log**_**2**_ **HE4**				2.23	1.60–3.10	** <0.001**	3.31	2.10–5.21^*^	** <0.001**
**FIGO Stage (>I vs. I)**				5.77	2.62–12.7	** <0.001**	3.73	1.80–7.75	**0.001**
**Age (years)**							1.64	1.00–2.67	**0.048**

* *Due to the non-linear relationship between log_2_ HE4 and PFS, the reported Hazard Ratio (HR) is for a 2 fold increase in the linear portion of the curve, namely HE4 from 64 to 128 pmol/L*.

*Bold numbers indicate a statistically significant difference with a p < 0.05*.

$ *log-rank test p-value*.

In multivariable analysis, a full model including the variables available for all patients (age, tumor diameter, FIGO stage, first-line treatment, and log_2_HE4) was set as a starting point. Using backward selection and LASSO, the variables that affect less on the model were progressively removed. A restricted model, accounting for HE4, FIGO stage, and age (for PFS only), remained in the final model as independent prognostic factors (Table 2). In order to evaluate the effect of excluding variables with many missing values on HE4 estimates (clinical tumor size, lymph-vascular space invasion, stromal infiltration, parametrial invasion, and lymph node status) in multivariable models, we performed multiple data imputation (*N* = 9 datasets) using MICE algorithm. On every imputed dataset we fitted an L1 penalized Cox model. We selected those variables that had a non-zero coefficient in at least five out of nine models. We then fit Cox models on an individual imputed dataset using the selected covariates: the final models for both OS and PFS included stage, age, clinical tumor size, lymph-vascular space invasion, stromal infiltration, parametrial invasion, and lymph node status. Pooled estimates for HE4 were substantially in line with those obtained in the single model (OS, HR = 2.09, CI_95%_ = 1.37–3.20, *p* < 0.001; PFS, HR = 2.28, CI_95%_ = 1.40–3.73, and *p* = 0.001).

## Discussion

In the present investigation, we demonstrated for the first time that high sHE4 levels are correlated with aggressive tumor characteristics and worse prognosis in UCC patients. Clinical evidence has confirmed the role of sHE4 as a biomarker with diagnostic and prognostic value in gynecological malignancies, mostly in ovarian and endometrial carcinoma (10–15). In particular, high sHE4 levels have been correlated with poor clinical outcome, as an independent prognostic factor. Herein, we investigated its role in UCC. Firstly, our study demonstrated that pre-treatment sHE4 levels were significantly elevated in UCC patients compared to healthy controls (63% sensitivity, 80% specificity), but the diagnostic performance of sHE4 does not exceed that of commonly used screening tests, such as HPV test or cervical smear cytology (23). Most importantly, we reported for the first time that higher sHE4 levels are correlated with aggressive tumor characteristics (advanced stage and persistence of disease after primary treatment; marginally, with high tumor grade and larger tumor size), and represented a prognostic marker for shorter OS and PFS in UCC patients, either in univariable or in multivariable survival analysis. Thus, we confirmed the independent prognostic impact of sHE4 levels in UCC, as already reported in ovarian and endometrial carcinoma by several groups, including ours (10, 11, 13, 14).

In previous investigations, clinico-pathological tumor characteristics such as histological grade, FIGO stage, and lymph node status have been reported as significant prognosticators for UCC outcome (4). Similarly, in our multivariable analysis, the FIGO stage confirmed its role as a prognostic factor, although clinical staging is known to be not as accurate as surgical staging (4). Pre-treatment tumor markers detected in serum may offer a simple and economic opportunity for preoperative prediction of the clinical course of the disease. The results of our study show that sHE4 levels can be an aid for clinical outcome prediction in UCC patients.

This finding can be considered an additional step toward the development of more useful tools with prognostic purposes and disease risk assessment in the pre-treatment period for UCC patients when important clinical decisions on surgical extension or therapy need to be taken.

Patient treatment heterogeneity is the main limitation of the present study. For the application in clinical practice, the prognostic significance of sHE4 will need to be confirmed on larger UCC cohorts homogeneous for the interventions received. This will allow to evaluate the predictive value of sHE4 in detecting the response to chemo or radiotherapies or the correlation with pathological characteristics after surgery.

To the best of our knowledge, only one previous study by Montagnana et al. (24) evaluated sHE4 levels in a small group of 14 UCC patients, as well as in women with benign and malignant pelvic masses. Serum HE4 levels were significantly higher in UCC patients than in healthy controls, but lower compared to ovarian cancer patients (24), in agreement with the current results and with our previous papers (12, 13). Diniz et al. (25) recently evaluated by immunohistochemistry HE4 tissue expression in UCC, demonstrating that intraepithelial carcinomas and normal cervical epithelia had low or negative HE4 expression, while invasive UCC presented increased HE4 positivity, regardless of histological subtypes, consistent with the results obtained at the serum level in the present study. Accordingly, analysis of HE4 protein levels in both serum and tissue suggests that HE4 is more expressed in aggressive UCC phenotypes.

Although the role of HE4 as a biomarker has been well-studied in clinical settings, its molecular and biological function has been investigated only in a few studies concerning ovarian and endometrial cancers (26–29).

HE4 overexpression was demonstrated to promote cancer cell proliferation, migration, invasion, and metastasis *in vitro* and to enhance tumor growth, metastasis rate, and platinum-chemoresistance *in vivo*, both in ovarian and endometrial cancer cell lines (26–29). The association of HE4 overexpression with epithelial ovarian cancer progression has been related to its effects on the EGFR-MAPK signaling pathway and ECM-receptor interaction pathway (26–28). Further investigations are still needed to clarify the molecular mechanisms involving HE4 in carcinogenesis and tumor progression, in particular concerning cervical cancer, for which functional studies do not currently exist.

In conclusion, the present investigation provides evidence that sHE4 could be a preoperative tumor biomarker of poor prognosis for UCC patients, and may be useful in treatment option choice. Serum HE4 could represent an additional tool for identifying UCC patients with a more aggressive form of the disease, although our findings should be further validated in prospective large-scale studies.

## Data Availability Statement

The raw data supporting the conclusions of this article will be made available by the authors, without undue reservation.

## Ethics Statement

The studies involving human participants were reviewed and approved by Comitato Etico di Brescia, ASST Spedali Civili, Brescia, Italy. The patients/participants provided their written informed consent to participate in this study.

## Author Contributions

EB, FO, and AR conceived and designed the study. LZ, CR, and PT performed the experiments. SC has been in charge of statistical analysis. GT, VZ, and DC collected data. EB, FO, and AR contributed to the analyses and interpretation of data. AR and EB wrote the first draft of the manuscript and all authors contributed to paper discussion and reviewed the manuscript. All authors have accepted responsibility for the entire content of this manuscript and approved its submission.

## Conflict of Interest

The authors declare that the research was conducted in the absence of any commercial or financial relationships that could be construed as a potential conflict of interest.

## Abbreviations

HE4: Human epididymis protein 4
sHE4: serum HE4
UCC: uterine cervical carcinoma
FC: Fold Change
HR: Hazard Ratio
CI_95%_: 95% confidence interval
LRT: likelihood ratio tests.

## References

[1] Jemal A Bray F Center MM Ferlay J Ward E Forman D Global cancer statistics. CA Cancer J Clin. (2011) 61:69–90. 10.3322/caac.20107 21296855

[2] Elit L. Cervical cancer in the older woman. Maturitas. (2014) 78:160–67. 10.1016/j.maturitas.2014.04.018 24861965

[3] Jürgenliemk-Schulz IM Beriwal S de Leeuw AAC Lindegaard JC Nomden CN Pötter R. Management of nodal disease in advanced cervical cancer. Semin Radiat Oncol. (2019) 29:158–65. 10.1016/j.semradonc.2018.11.002 30827454

[4] Cohen PA Jhingran A Oaknin A Denny L. Cervical cancer. Lancet. (2019) 393:169–82. 10.1016/S0140-6736(18)32470-X 30638582

[5] Duffy MJ. Evidence for the clinical use of tumour markers. Ann Clin Biochem. (2004) 41:370–77. 10.1258/0004563041731529 15333188

[6] Huang G Chen R Lu N Chen Q Lv W Li B. Combined evaluation of preoperative serum CEA and CA125 as an independent prognostic biomarker in patients with early-stage cervical adenocarcinoma. Onco Targets Ther. (2020) 13:5155–64. 10.2147/OTT.S250614 32606736 PMC7292260

[7] Liu Z Shi H. Prognostic role of squamous cell carcinoma antigen in cervical cancer: a meta-analysis. Dis Markers. (2019) 2019:6710352. 10.1155/2019/6710352 31275450 PMC6589214

[8] Bingle L Singleton V Bingle CD. The putative ovarian tumour marker gene HE4 (WFDC2) is expressed in normal tissues and undergoes complex alternative splicing to yield multiple protein isoforms. Oncogene. (2002) 21:2768–73. 10.1038/sj.onc.1205363 11965550

[9] Kirchhoff C Habben I Ivell R Krull N. A major human epididymis-specific cDNA encodes a protein with sequence homology to extracellular proteinase inhibitors. Biol Reprod. (1991) 45:350–57. 10.1095/biolreprod45.2.350 1686187

[10] Simmons AR Baggerly K Bast RC Jr. The emerging role of HE4 in the evaluation of epithelial ovarian and endometrial carcinomas. Oncology. (2013) 27:548–56. 23909069 PMC4085777

[11] Bignotti E Ragnoli M Zanotti L Calza S Falchetti M Lonardi S. Diagnostic and prognostic impact of serum HE4 detection in endometrial carcinoma patients. Br J Cancer. (2011) 104:1418–25. 10.1038/bjc.2011.109 21468050 PMC3101927

[12] Ruggeri G Bandiera E Zanotti L Belloli S Ravaggi A Romani C. HE4 and epithelial ovarian cancer: comparison and clinical evaluation of two immunoassays and a combination algorithm. Clin Chim Acta. (2011) 412:1447–53. 10.1016/j.cca.2011.04.028 21557935

[13] Bandiera E Romani C Specchia C Zanotti L Galli C Ruggeri G. Serum human epididymis protein 4 and risk for ovarian malignancy algorithm as new diagnostic and prognostic tools for epithelial ovarian cancer management. Cancer Epidemiol Biomarkers Prev. (2011) 20:2496–506. 10.1158/1055-9965.EPI-11-0635 22028406 PMC3237732

[14] Zanotti L Bignotti E Calza S Bandiera E Ruggeri G Galli C. Human epididymis protein 4 as a serum marker for diagnosis of endometrial carcinoma and prediction of clinical outcome. Clin Chem Lab Med. (2012) 50:2189–98. 10.1515/cclm-2011-0757 23096757

[15] Vezzoli M Ravaggi A Zanotti L Miscioscia RA Bignotti E Ragnoli M. RERT: A novel regression tree approach to predict extrauterine disease in endometrial carcinoma patients. Sci Rep. (2017) 7:10528. 10.1038/s41598-017-11104-4 28874808 PMC5585365

[16] Hertlein L Stieber P Kirschenhofer A Krocker K Nagel D Lenhard M. Human epididymis protein 4 (HE4) in benign and malignant diseases. Clin Chem Lab Med. (2012) 50:2181–8. 10.1515/cclm-2012-0097 23093276

[17] Huber PJ Robust Statistics. New York, NY: Wiley (1981).

[18] Grambsch P Therneau T Proportional hazards tests and diagnostics based on weighted residuals. Biometrika. (1994) 81:515–26. 10.1093/biomet/81.3.515

[19] Simon N Friedman J Hastie T Tibshirani R. Regularization paths for cox's proportional hazards model via coordinate descent. J Stat Softw. (2011) 39:1–13. 10.18637/jss.v039.i05 27065756 PMC4824408

[20] Harrell FE Regression Modeling Strategies: With Applications to Linear Models, Logistic Regression, and Survival Analysis. New York, NY: Springer-Verlag New York, Inc (2010).

[21] Van Buuren S Flexible Imputation of Missing Data, 2nd edn. Boca Raton, FL: Chapman & Hall/CRC (2018).

[22] Hothorn T Zeileis A. Generalized maximally selected statistics. Biometrics. (2008) 64:1263–69. 10.1111/j.1541-0420.2008.00995.x 18325074

[23] Mustafa RA Santesso N Khatib R Mustafa AA Wiercioch W Kehar R. Systematic reviews and meta-analyses of the accuracy of HPV tests, visual inspection with acetic acid, cytology, and colposcopy. Int J Gynaecol Obstet. (2016) 132:259–65. 10.1016/j.ijgo.2015.07.024 26851054

[24] Montagnana M Lippi G Ruzzenente O Bresciani V Danese E Scevarolli S. The utility of serum human epididymis protein 4 (HE4) in patients with a pelvic mass. J Clin Lab Anal. (2009) 23:331–35. 10.1002/jcla.20340 19774626 PMC6649112

[25] Diniz G Karadeniz T Sayhan S Akata T Aydiner F Ayaz D. Tissue expression of human epididymal secretory protein 4 may be useful in the differential diagnosis of uterine cervical tumors. Ginekol Pol. (2017) 88:51–55. 10.5603/GP.a2017.0011 28326512

[26] Lu R Sun X Xiao R Zhou L Gao X Guo L. Human epididymis protein 4 (HE4) plays a key role in ovarian cancer cell adhesion and motility. Biochem Biophys Res Commun. (2012) 419:274–80. 10.1016/j.bbrc.2012.02.008 22342977

[27] Moore RG Hill EK Horan T Yano N Kim K MacLaughlan S. HE4 (WFDC2) gene overexpression promotes ovarian tumor growth. Sci Rep. (2014) 4:3574. 10.1038/srep03574 24389815 PMC3880958

[28] Zhu L Zhuang H Wang H Tan M Schwab CL Deng L. Overexpression of HE4 (human epididymis protein 4) enhances proliferation, invasion and metastasis of ovarian cancer. Oncotarget. (2016) 7:729–44. 10.18632/oncotarget.6327 26575020 PMC4808029

[29] Li J Chen H Mariani A Chen D Klatt E Podratz K. HE4 (WFDC2) promotes tumor growth in endometrial cancer cell lines. Int J Mol Sci. (2013) 14:6026–43. 10.3390/ijms14036026 23502467 PMC3634435

