# Supplementary figures and images for: Pre-treatment Serum HE4 Level as a Novel Independent Prognostic Biomarker for Uterine Cervical Carcinoma Patients
Source: Front Oncol. 2020 Sep 29;10:584022. doi: 10.3389/fonc.2020.584022 (PMC7550621; doi:10.3389/fonc.2020.584022)

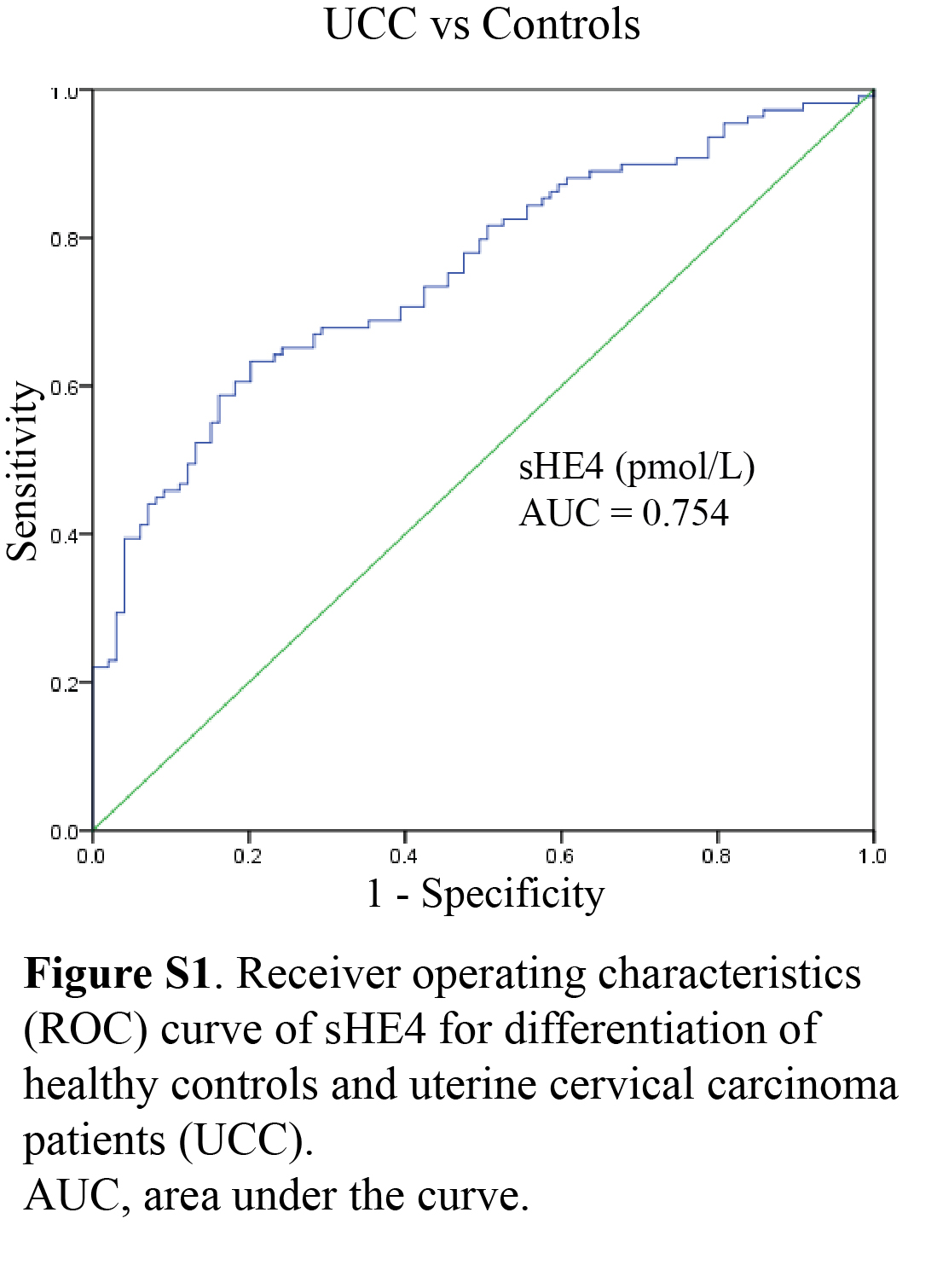

Supplement: Supplementary file 2 [file Image_1.JPEG]
